# Presence of *Candida* cell wall derived polysaccharides in the sera of intensive care unit patients: relation with candidaemia and *Candida* colonisation

**DOI:** 10.1186/cc13953

**Published:** 2014-06-29

**Authors:** Julien Poissy, Boualem Sendid, Sébastien Damiens, Ken Ichi Ishibashi, Nadine François, Marie Kauv, Raphaël Favory, Daniel Mathieu, Daniel Poulain

**Affiliations:** 1 Université Lille Nord de France, 1 rue Lefèvre, 59000 Lille, France; 2 INSERM U995-2, Faculté de Médecine-Pôle recherche, Place de Verdun, 59045 Lille, France; 3 Pôle de Réanimation, CHRU Lille, 2 avenue Oscar Lambret, 59037 Lille Cedex, France; 4 Laboratoire de Parasitologie Mycologie, Institut de Microbiologie, Pôle de Biologie Pathologie Génétique, rue Paul Nayrac, CHRU Lille, 59037 Lille Cedex, France; 5 Laboratory for Immunopharmacology of Microbial Products, School of Pharmacy, University of Pharmacy and Life Science, Hachioji, Tokyo 192-0392, Japan

## Abstract

**Introduction:**

Prompt diagnosis of candidaemia and invasive candidosis is crucial to the early initiation of antifungal therapy. The poor sensitivity of blood cultures (BCs) has led to the development of fungal glycan tests as a diagnostic adjunct. We analysed the performance of tests for the detection of circulating β-D-1,3-glucan (BDG) and mannan in the intensive care unit (ICU) setting.

**Methods:**

This retrospective, case–control study included 43 ICU patients with candidaemia and 67 controls, hospitalised on the same ward and assessed weekly for yeast colonisation with simultaneous serum sampling; 340 sera taken before and after positive BCs were available for the cases group and 203 for the controls. BDG and mannan levels were determined using the Fungitell^®^ and Platelia™ Candida Ag tests, respectively.

**Results:**

BDG was detected early in sera from cases patients but was also present in several sera from controls. Increasing the cut-off from 80 pg/mL to 350 pg/mL and 800 pg/mL resulted in sensitivity/specificity ratios of 0.97/0.31, 0.65/0.74, 0.30/0.86, respectively. Detection of mannan was more specific but lacked sensitivity. No obvious correlation was found between BDG and colonisation, but a trend existed between high colonisation and high BDG. Candidaemia relapses were associated with a rise in BDG and mannan but, in contrast to the transient nature of mannan, BDG persisted up to 7 weeks after positive BCs.

**Conclusion:**

A combination of mannan and BDG tests could be used to guide pre-emptive therapeutic decisions in ICU patients.

## Introduction

Invasive candidosis (IC) is one of the leading causes of nosocomial infection and *Candida* species rank fourth among the pathogens involved in bloodstream infections [1]. Despite current progress in research and antifungal therapy, the incidence and attributable mortality of candidaemia remain high [2] due to difficulties in the establishment of an accurate and early diagnosis. In the ICU, candidaemia has a prevalence of 7/1,000 patients, with an attributable mortality of >40% compared with 30% for bacteraemia [3]. Mortality increases from 10% if antifungal therapy is introduced within 12 hours of the onset of candidaemia to 35% when treatment is initiated more than 48 hours after [4]. These figures are worse in cases of septic shock due to *Candida* species [5]. The challenge is therefore to manage the delay in initiation of antifungal treatment, especially as 50% of cases of IC are not detected by blood cultures (BCs) and 48 hours are generally required for yeast isolation [6]. This low sensitivity of BCs was observed in several large postmortem studies evaluating the sensitivity of BCs for the diagnosis of deep-seated *Candida* invasion [7] and was shown to range from 28% in cases of single organ candidosis to 58% in cases of disseminated IC [8]. Improvement of BC systems has only decreased the delay in yeast isolation for certain species without any improvement in the sensitivity [9]. Relying on BCs or waiting for BC results is thus not appropriate for managing patients at high risk of IC.

Considering the need for alternatives to BCs for early diagnosis, the Infectious Diseases Society of America and the European Society of Clinical and Microbiology and Infectious Diseases have recommended the use of nonculture-based methods to help make therapeutic decisions [10,11]. Among the surrogate markers, some *Candida* cell-wall-derived polysaccharides or oligosaccharides resulting from their catabolism (glycans) can be detected in the sera of patients with candidosis. These consist of mannan, a polymer of mannose representing the polysaccharide moiety of molecules from the outer cell wall layers, and β-d-1,3-glucan (BDG), a polymer of glucose making up the fibrils in the middle layers. The combined detection of glycan biomarkers and anti-mannan antibodies was also recommended in the last Surviving Sepsis Campaign for documentation of the microorganisms involved in septic shock [12]. Numerous studies have evaluated mannan and BDG detection tests for the diagnosis of IC in patients with haematological malignancies and in surgical ICU patients; however, information about the value of glucanaemia and mannanaemia monitoring is scarce.

In this study, we looked at ICU patients with candidaemia and control patients from the same ward and with the same high-risk factors/predisposing conditions for IC with the aim of analysing BDG and mannan levels during hospitalisation in relation to candidaemia onset or *Candida* colonisation. The primary evaluation measure was an assessment of the two tests to make an early diagnosis of candidaemia. In addition, we analysed how these biomarkers could predict candidaemia relapses or a favourable outcome. Finally, we propose a biomarker-based algorithm designed especially for the management of ICU patients, most of whom are at high risk of IC.

## Materials and methods

### Patients

This retrospective, case–control study involved adult patients hospitalised in a 50-bed polyvalent ICU department in a tertiary university teaching hospital. The database of the clinical mycology laboratory was screened to select patients with a positive BC for *Candida* over the period 2005 to 2010. We focused on patients >18 years old for whom sera were available at least 1 week before and 1 week after the day of candidaemia. The control group consisted of patients hospitalised on the same ward with *Candida* colonisation but no evidence of IC; five body sites (urine, anal swabs, nasal swabs, throat and tracheal aspirates when patients were intubated) were sampled once a week for the semi-quantitative determination of yeast colonisation. The medical files for these patients were analysed retrospectively using a standardised questionnaire to look for arguments for IC based on the criteria previously used by Mohr and colleagues [13] and derived from the European Organisation for Research and Treatment of Cancer/Mycoses Study Group criteria [14]. We also looked for evidence of invasive aspergillosis and infection by *Pneumocystis jirovecii* and excluded patients who had criteria for these two opportunistic fungal infections.

### Blood cultures

BCs were performed by drawing 10 ml blood from either the peripheral vein or arterial catheters into Mycosis ICF vials incubated at 37°C for up to 7 days in a Bactec FX System (Becton Dickinson, Le Pont de Claix, France).

### Measurement of β-d-1,3-glucan in serum

BDG in serum was measured using the Fungitell^®^ kit (Associates of Cape Cod Inc., Falmouth, MA, USA), following the manufacturer’s instructions. The recommended cutoff value of 80 pg/ml was used to define positivity. Samples with BDG levels >500 pg/ml were diluted and retested.

### Measurement of mannan antigen and anti-mannan antibodies in serum

Mannan antigen and anti-mannan antibodies were measured using the Platelia™ Candida Ag (mannan) and Platelia™ Candida Ab (mannan Ab) tests (Bio-Rad, Marnes la Coquette, France) according to the manufacturer’s instructions. The recommended cutoff values for the mannan and mannan Ab tests used between 2005 and 2010 were 0.5 ng/ml and >10 AU, respectively. Samples with mannan >500 pg/ml were diluted and retested.

### Intensity of colonisation

Colonisation intensity was determined for each date of sampling in the control group. Each sample was incubated on Chromagar^®^ (Becton Dickinson, Heidelberg, Germany) medium under standard conditions. Presumptive identification was confirmed by *ad hoc* tests (Bichrolatex and glabrata RTT, Fumouze Diagnostics, Levallois-Perret, France; API 20C, Biomérieux France, Craponne) and the number of colony-forming units was scored as follows: score 1, <10 colony-forming units; score 2, 10 to 50 colony-forming units; score 3, >50 colony-forming units; score 4, >50 colony-forming units confluent. Intensity of colonisation was determined for each date of sampling, by dividing the sum score for each colonised site by the number of sites sampled giving a mean *Candida* load. An overall score of >4 was possible in the case of isolation of several *Candida* species because we added together the colonisation intensity for each species.

For the patients with candidaemia, data on colonisation were derived from a systematic weekly survey of urine, nasal, tracheal and anal colonisation for the isolation of multiresistant bacteria, where yeasts were also isolated.

### Statistical analysis

Quantitative variables are expressed as median values and 95% confidence intervals. Qualitative variables were analysed using Fisher’s exact test and quantitative variables using the Wilcoxon test. Tests were two-tailed. Pearson’s correlation test was performed when necessary. A significance threshold of 0.05 was retained. All statistical analyses were performed using EpiInfo V3.5.3 (Centers for Disease Control and Prevention). Graphics were drawn using Graphpad Prism6 (GraphPad Software, San Diego, California).

### Ethical statement

All sera used in this study were sampled from patients followed in Lille University Hospital. When no results were available from routine tests, BDG and mannan levels were determined retrospectively from the residual frozen samples. No additional sampling was necessary. As sera were taken from a registered biological collection, patient consent was not required according to French law. Institutional review board approval was given by the Comité de Protection des Personnes Nord-Ouest IV, the ethical committee of our institution.

## Results

### Description of the study population

A total of 117 patients with candidaemia were identified during the study period; 43 (36.8%) of these had all of the criteria for inclusion in the study. Clinical and demographic data for the candidaemia and control groups are presented in Table 1. The groups did not differ in terms of demography, Simplified Acute Physiology Score and risk factors for *Candida* infection, except for intestinal surgery, exclusive parenteral nutrition and extrarenal epuration. A significant difference in mortality was recorded during both the ICU stay and the subsequent hospital stay.

**Table 1:** Major characteristics of patients

**Characteristic**	**Cases**	**Controls**	***P *** **value**
	**(*n*** **= 43)**	**(*n*** **= 67)**	**(two-tailed)**
Age (years)	61.0 (50.0 to 72.5)	60.5 (49.5 to 70.0)	0.76
Sex ratio (male/female)	2.9	2.3	0.67
Simplify Acute Physiology Score	51.5	53.0	0.23
Surgical patient	37.2	20.3	0.07
Abdominal surgery	30.2	10.5	0.01
Other surgery	7.0	10.5	0.7
Deep venous catheter	97.7	96.8	1
Broad-spectrum antibiotherapy	100	98.4	1
Corticoids	30.2	39.7	0.41
Vasopressor therapy	65.1	57.8	0.55
Exclusive parenteral nutrition	37.2	4.7	<5 × 10^-5^
Extrarenal epuration	62.8	29.7	0.001
Mechanical invasive ventilation	100	98.4	1
Duration of mechanical ventilation (days)	31.5 (14.0 to 47.0)	28.5 (15.5 to 42.0)	0.5
Neutropaenia	7.0	6.4	1
Antifungal therapy	86.0	18.0	<5 × 10^-5^
Fluconazole	59.5	54.5	
Voriconazole	13.5	0	
Caspofungin	54.1	54.6	
Liposomal amphotericin-B	5.4	9.1	
5-Fluorocytosine	2.7	0	
Bacteraemia	58.1	27.0	0.002
Distribution of bacteria			
Gram-positive	28.0	18.8	
Gram-negative	56.0	56.2	
Mixed Gram-positive and Gram-negative	16.0	25.0	
ICU duration of hospitalisation	33.5 (23.5 to 60.0)	29.0 (19.5 to 46.5)	0.5
ICU mortality	62.8	38.5	0.02
Hospital mortality	65.1	44.6	0.05

Quantitative data are expressed as mean (25th to 75th quartile). Qualitative data are expressed as a percentage.

The median delay between admission to the ICU and appearance of candidaemia was 19 days (range: 10 to 31 days). Only one *Candida* species was found in BCs (*Candida albicans* in 40.5%, *Candida parapsilosis* in 23.8%, *Candida tropicalis* in 19%, *Candida glabrata* in 16.7%), except for one patient who had both *C. albicans* and *C. parapsilosis*. Weekly microbial surveillance of these patients revealed that all were colonised by *Candida* species. For the control patients, weekly multisite determination of yeast colonisation revealed that all except two patients were colonised with *Candida* species and approximately one-third was colonised with more than one species: two species in 15 patients, three species in eight patients, and four species in two patients. The relative prevalence of the different *Candida* species was similar to that in the candidaemia group (in decreasing order: *C. albicans*, *C. parapsilosis*, *C. glabrata*, *C. tropicalis*).

### Glucanaemia and mannanaemia during the ICU stay

**Figure 1:**
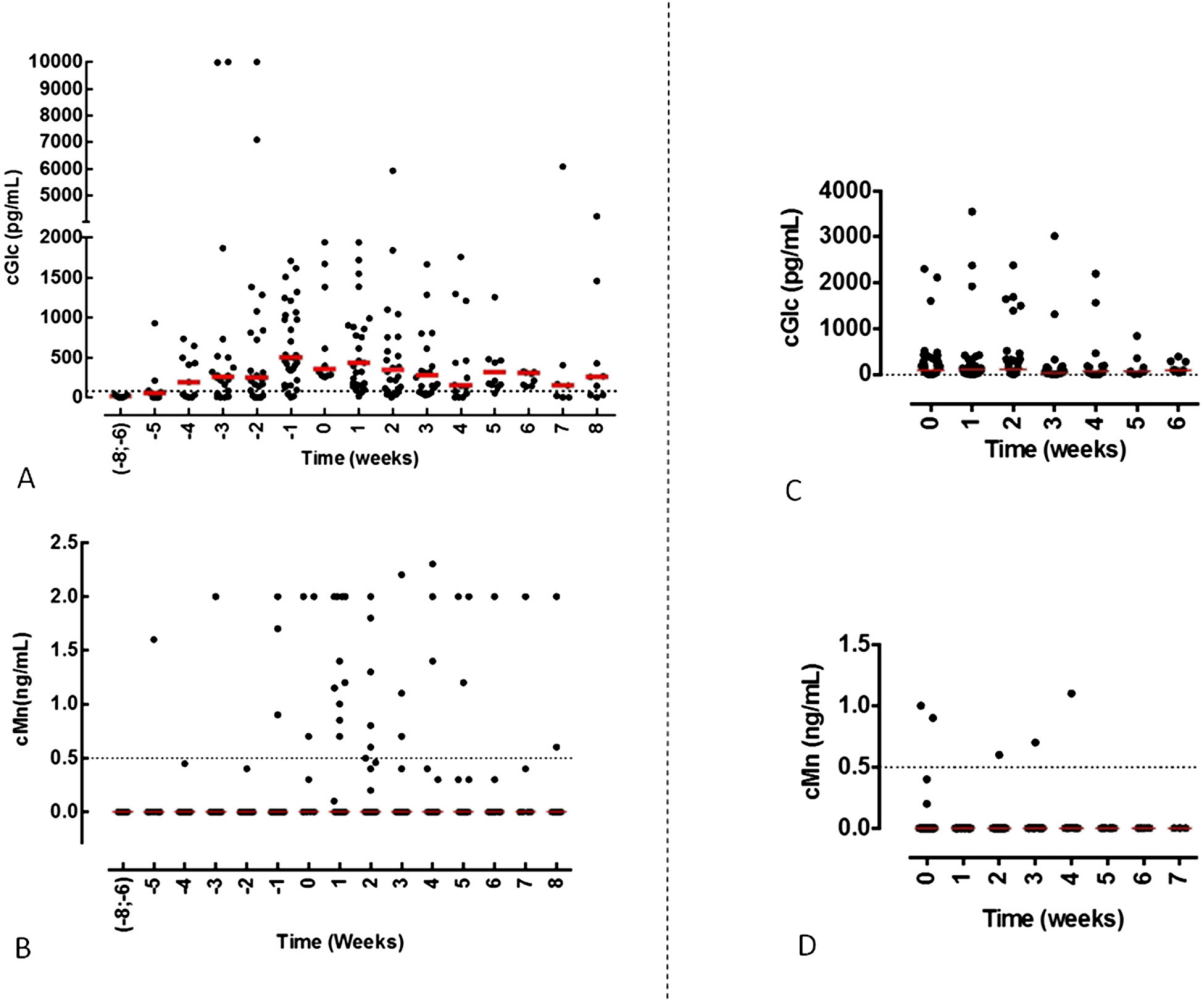
**Kinetics of β-d-1,3-glucan and mannan in candidaemic patients and controls.** Levels of **(A)** β-d-1,3-glucan (BDG) and **(B)** mannan were measured in 341 sera available from 43 patients with positive blood cultures. Sera were collected as a function of the number of weeks of hospitalisation in the ICU, with D0 as the day on which the first positive blood culture was taken. Levels of **(C)** BDG and **(D)** mannan were measured in 203 sera available from 67 control patients hospitalised on the same ward as a function of number of weeks of hospitalisation (control group). cGLC, concentration of BDG; cMan, concentration of mannan.

Glucanaemia and mannanaemia in sera from patients with candidaemia are shown in Figure 1A and 1B, respectively, as a function of weeks of hospitalisation before and after positive BCs. Glucanaemia was observed several weeks before positive BC. The median delay between positive BDG and positive BC was 10 days. The glucan level was maximal the week before positive BC. At the date of positive BC, all sera/patients had BDG levels >250 pg/ml. A global decrease in BDG was then observed after week 3, although BDG persisted in some patients for up to 8 weeks.

The data were analysed in terms of sensitivity, specificity and likelihood ratios with reference to the kinetics of BDG. The results are presented in Table 2 for the whole period of serum collection. To respond to the questions ‘How can glycanaemia predict the onset of IC?’ and ‘How can glycanaemia reveal IC?’ we considered the period from day 7 before to day 7 after the first positive BC. This analysis was also carried out for patients with negative BCs.

**Table 2:** Sensitivity, specificity and likelihood ratios for glucanaemia and mannanaemia

	**Sensitivity**		**Specificity**		**Positive LR**		**Negative LR**	
	**Week -8/+8**	**Day –7/+7**	**Week -8/+8**	**Day –7/+7**	**Week -8/+8**	**Day –7/+7**	**Week -8/+8**	**Day –7/+7**
β-d-1,3-glucan	100	97.1	30.6	30.6	1.4	1.4	0	0.1
Mannan	38.5	32.3	95.8	95.8	9.2	7.7	0.6	0.7
Mannan antibody	58.1	52.9	66.2	66.2	1.7	1.6	0.6	0.7
Mannan or mannan antibody	79.5	58.8	66.2	64.8	2.4	1.7	0.3	0.6

Sensitivity, specificity and positive and negative likelihood ratios (LRs) for glucanaemia (β-d-1,3-glucan), mannanaemia and anti-mannan antibody detection tests, as well as a combination of mannan/mannan antibody, during the whole period of serum collection (week 8 before to week 8 after the first positive blood culture (Week -8/+8)), and the critical period for diagnosis (day 7 before to day 7 after the first positive blood culture (Day –7/+7)).

**Figure 2:**
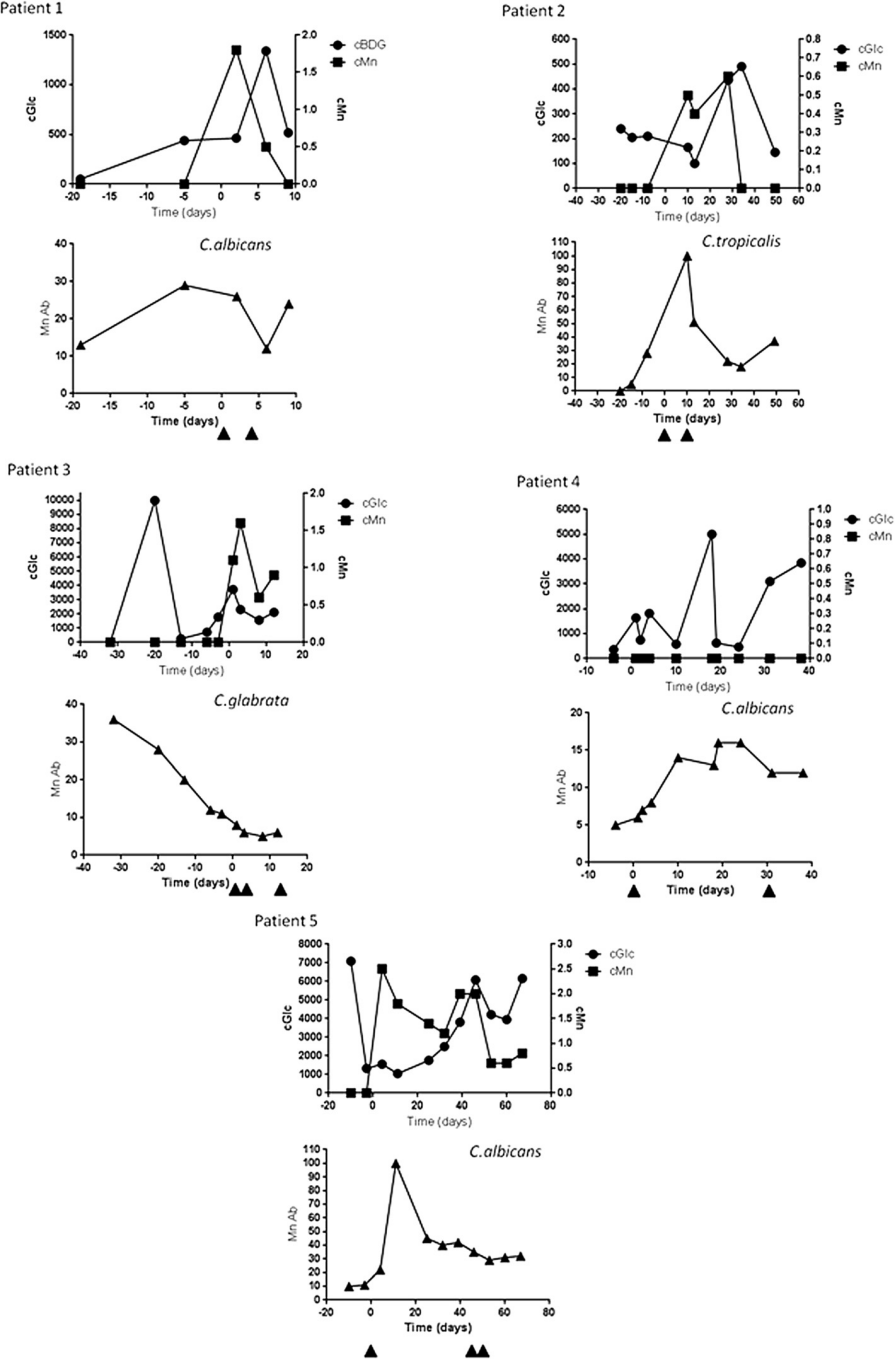
**Kinetics of β-d-1,3-glucan, mannan and mannan antibody in five patients with candidaemia relapses.** The dates of subsequent *Candida* isolations from blood are indicated by arrows. All patients, except Patient 4 with no detectable mannan, had values above the cutoff point for all tests, but with different kinetics. β-d-1,3-glucan (BDG) and mannan presented different kinetics of circulation in a single patient. The well-known transient nature of circulating mannan was observed and correlated with an inverse evolution of mannan antibody (Patients 1, 2, 3 and 5). Interestingly a sharp decrease in BDG was also observed over a short period of time (Patients 3, 4 and 5). cGLC/cBDG, concentration of BDG; cMn, concentration of mannan; Mn Ab, concentration of mannan antibody.

We then analysed how glycanaemia could predict relapse or a favourable outcome by focusing on the 13 patients who had several episodes of candidaemia. Patients were divided into two groups: those with the continuous isolation of yeasts from blood with intervals of <48 hours (two BCs for two patients, three BCs for four patients, five BCs for one patient and nine BCs for one patient); and patients with well-separated candidaemia episodes corresponding to relapses (*n* = 5). The BDG and mannan Ab profiles of these patients are shown in Figure 2 and their clinical evolution is described in Additional file 1. This panel of patients is representative of the diversity of conditions encountered in the ICU and susceptibility to IC.When analysing the kinetics of BDG, mannan and mannan Ab in Figure 2, all patients (except Patient 4 with no detectable mannan) had values above the cutoff point for all tests, but with different evolutions. There were positive slopes for BDG and mannan before secondary candidaemia, but different kinetics of circulation in a single patient. The well-known transient circulation of mannan was observed and correlated with the inverse evolution of mannan Ab (Patients 1, 2, 3 and 5). Interestingly, a sharp decrease in BDG was observed in some patients over a short period of time (see Patients 3, 4 and 5).

Besides the patients described above, the other 25 patients had no documented clinical or mycological signs of relapse. The duration of the survey ranged from day 6 to day 102. At the end of the survey, 21 patients still had BDG above the cutoff value and only four had BDG below the cutoff value. When considering the evolution of the BDG curve, a negative slope was observed for 10 patients, including two who were below the cutoff values at day 9 and day 86, and two who became negative at day 71 and day 74; all other patients still had BDG >150 pg/ml. Two patients had a positive slope close to BC isolation (final points at 310 pg/ml and 760 pg/ml at day 6 and day 10, respectively). The other patients (*n* = 13) had stable BDG levels above the cutoff value, including levels as high as 2,223 pg/ml, 1,030 pg/ml and 1,458 pg/ml at day 7, day 12 and day 47, respectively. Median BDG and median duration of the survey were 314 pg/ml and 18 days, respectively.

### Patients hospitalised in the ICU with no evidence of *Candida* infection

The results of glucanaemia and mannanaemia tests for the control group are shown in Figure 1C and 1D, respectively, according to the duration of hospitalisation (weeks). As early as the first week of hospitalisation, about 50% of the patients presented at least one serum sample with BDG above the cutoff value. This percentage remained stable during the following weeks, up to more than 1 month of hospitalisation. By contrast, only five (2%) sera samples taken from three different patients had mannanaemia above the cutoff value, and this was transient for all of them.

For the patients followed for *Candida* colonisation during hospitalisation, glucanaemia was assessed as a function of *Candida* load at the time of serum sampling. There was no correlation between BDG and yeast load in these patients and 17 (8.4%) sera samples had values over 1,000 pg/ml (Additional file 2). As shown in Additional file 3, BDG was significantly associated with a high global *Candida* load. Analysis of the effect of colonisation on mannan did not show any correlation for the very limited number (*n* = 3) of control patients with mannanaemia.

### Impact of variation of the cutoff value on the performance of β-d-1,3-glucan and mannan detection

**Figure 3:**
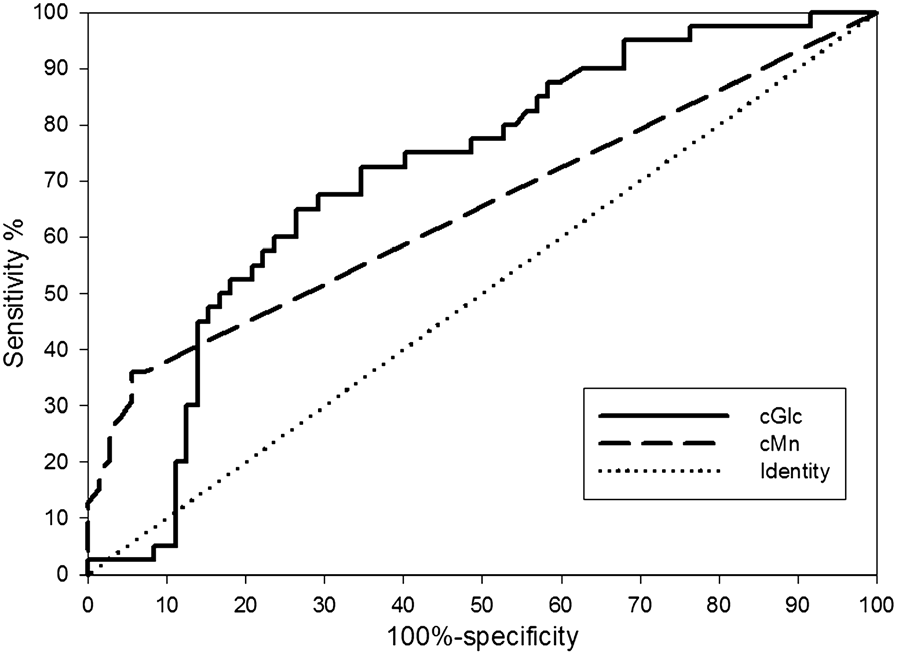
**Receiver operating characteristic curves for β-d-1,3-glucan (plain line) and mannan (dotted line) for the period from day 7 before to day 7 after the first positive blood culture**. Area under the curve (95% confidence interval) for β-d-1,3-glucan (BDG) = 0.71 (0.61 to 0.81) with *P* < 0.0001; threshold for the best sensitivity/specificity ratio, 353 (0.65 to 0.74). Area under the curve (95% confidence interval) for mannan = 0.63 (0.52 to 0.74) with *P* = 0.009; threshold for the best sensitivity/specificity ratio, 0.2 (0.36 to 0.94). When considering how the two tests could complement each other, mannan has an important contribution to specificity for low BDG values while higher BDG values have improved sensitivity and specificity. cGLC, concentration of BDG; cMn, concentration of mannan.

Receiver operating characteristic curves for BDG and mannan are shown in Figure 3 for the period from day 7 before to day 7 after the first positive BC. For BDG, for a cutoff value of 1,600 pg/ml, sensitivity was 0.05 with a specificity of >0.9. For a cutoff value of 800 pg/ml, the respective values were 0.30 and 0.86. The best sensitivity/specificity ratio (0.65/0.74) was obtained for a cutoff value of 350 pg/ml. For mannan, the best sensitivity/specificity ratio was 0.36/0.94 for a cutoff value of 0.2 ng/ml. From these results it appears that these two tests could be use to complement each other: mannan has an important contribution to specificity for low BDG values, while high BDG levels have improved sensitivity and specificity.

## Discussion

In this study, we evaluated the diagnostic value of glucanaemia in combination with mannanaemia in sera taken sequentially from ICU patients with candidaemia. In parallel, we also assessed the effect of *Candida* colonisation on serum levels of *Candida*-derived polysaccharides in patients from the same ICU department who did not have candidaemia. Altogether, 543 sera taken from 110 patients were analysed. The method of inclusion was intended to mimic clinical practice where clinicians have to quickly identify any ICU patients needing antifungal therapy, including those with negative BCs [6].

Glucanaemia was detected several days, or in some cases weeks, before the isolation of *Candida* species from the blood. In a pilot study evaluating plasma BDG measurement for the diagnosis of deep-seated mycoses and fungal febrile episodes, Obayashi and colleagues reported a sensitivity of 90% for a cutoff point of 20 pg/ml [15]. However, subsequent studies gave disparate results from different colorimetric and turbidimetric methods [16] using glucans as standards, in the absence of knowledge regarding the nature of the molecule(s) detected [17]. With the Fungitell test, the multicentre evaluation by Ostrosky-Zeichner and colleagues, including 107 cases of proven candidosis, reported a sensitivity/specificity ratio of 0.60/0.92 for a cutoff point of 80 pg/ml, although their use of control sera from healthy subjects could have led to an overestimation [18]. Using hospitalised patients from a large hospital study as controls, a similar sensitivity but lower specificity (70%) was reported [19]; this decreased further in another study involving surgical patients [13].

Impressive sensitivity and specificity (around 0.93) for BDG detection for the diagnosis of candidaemia were reported in septic patients with the early stages of fever [20]. This contrasts with the lower performance of this test reported in other studies [19,21], especially low specificity [22]. Indeed, studies published to date report a wide range of sensitivities and specificities for BDG detection [23]. The impact of neutropaenia [24] on BDG circulation is probably worth considering. However, in our cohort, an analysis of sensitivity performed on data collected from patients transferred from a haematology ward suggested that the performance of BDG detection was equivalent to that in non-neutropaenic patients (data not shown). Altogether, the best sensitivity/specificity ratio (0.65/074) for BDG was found for a cutoff value of 350 pg/ml, which is very close to that proposed by Jaijakul and colleagues for predictive efficiency [25].

This study clearly shows that the decrease in BDG over the course of the disease was much slower than the decrease in mannan. The mechanism involved in the catabolism of β-glucans may involve antibodies participating in their clearance since the existence of human anti-β-glucan antibodies has now been established [26,27], as well as an increase in these antibodies during candidaemia [28]. A second hypothesis for the difference in persistence of BDG and mannan is serum mannosidases that cause natural degradation/turnover of endogenous components; this is not the case for glucans, which, like glucuronoxylomannan of *Cryptococcus neoformans*, are resistant to degradation and are purely exogenous in nature [29]. From a clinical point of view, the slow decrease in BDG that we observed may limit its use for managing antifungal treatment, a conclusion that contradicts a previous study which proposed the follow-up of glucanaemia kinetics as a tool to evaluate the efficacy of echinocandin therapy [25]. However, some patients with candidaemia enrolled in our study had a second increase from their baseline BDG, associated with high levels of mannan before secondary BCs, suggesting that monitoring of glucanaemia and mannanaemia in patients receiving antifungal therapy is a useful strategy to identify patients with relapses.

The observation of persistently high BDG levels also raises the question of their impact on modulation of the immune response via their interaction with membrane and soluble Pattern Recognition Receptors [30].

In the control group, quantitative snapshot analysis did not reveal a correlation between BDG and yeast burden. However, analysis of cumulative colonisation revealed a trend for an association with BDG >1,000 pg/ml (data shown in Additional file 1).

In contrast to BDG, only three control patients had detectable mannan, in agreement with previous conclusions that the detection of mannan with the Platelia test has limited sensitivity but high specificity [11-13]. Here, the unusually high frequency of candidaemia episodes involving *C. parapsilosis* may also contribute to low sensitivity observed. In contrast to the other more pathogenic *Candida* species, the mannan epitope detected by the Platelia test is poorly expressed on *C. parapsilosis*[11]. As in a recent study [31], exclusion of *C. parapsilosis* resulted in a moderate gain in sensitivity (from 38% to 45%). Surprisingly, the specificity of BDG detection was also affected by the exclusion of *C. parapsilosis* cases (from 30% to 45%). This is of interest in view of the steady increase in incidence of *C. parapsilosis* candidaemia [32].

The low sensitivity of mannan detection has been attributed to the circulation of high levels of circulating mannan Ab and soluble lectins such as mannan-binding lectin forming immune complexes responsible for the rapid clearance of mannan [33-35]. This observation has led to the recommendation for repeated serum sampling and combined mannan and mannan Ab screening to improve the overall sensitivity of mannan-based diagnosis [36]. This recommendation has never been reevaluated, but it is interesting to observe here once again the relation of the respective slopes where a sharp decrease in mannan Ab is often predictive of a mannan peak and *vice versa*. However, mannan gave the higher positive likelihood ratio during the crucial period from day 7 before to day 7 after the first positive BC (7.7 vs. 1.4 for BDG).

This study has several limitations, related to its retrospective character and possible selection bias linked to the availability of sera from candidaemia patients and the constitution of the control group. Nevertheless, this latter group was representative of the high colonisation levels encountered in ICU patients, as reported in studies involving surgical ICU patients [37] and medical ICU patients who showed an increase in colonisation as a function of the duration of hospitalisation and cumulative exposure to risk factors [38]. This intense colonisation is itself a risk factor for the development of IC. Indeed, application of Mohr and colleagues’ criteria [13] led us to exclude a high proportion of patients from the control cohort, defined as having possible or probable IC. In our opinion, once this selection was made this group represented appropriate controls for ICU daily practice in order to evaluate the performance of biomarkers at discriminating the transition from colonisation to infection, which concerns up to one patient in 10. The differences between the two populations reported in Table 1 probably reflect both the background and the impact of IC [37,38].

**Figure 4:**
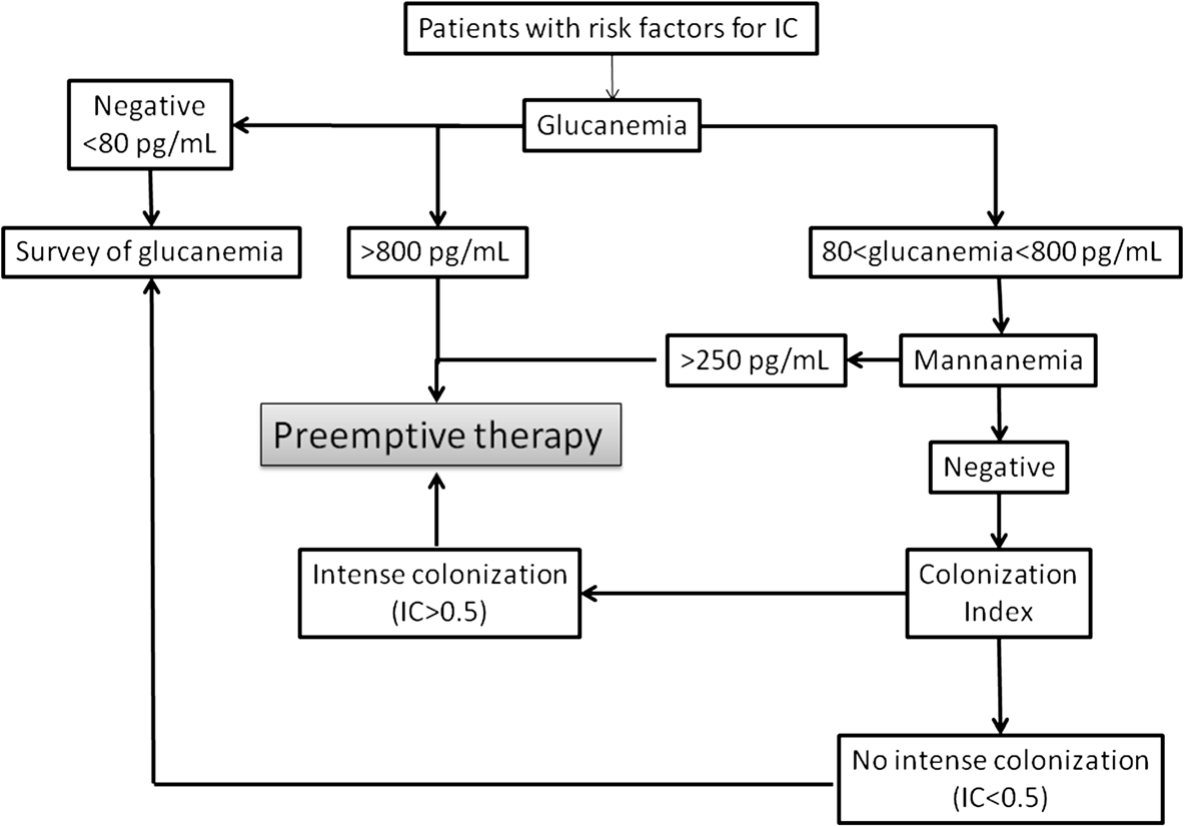
**Biomarker-based algorithm proposed for managing preemptive therapy in ICU patients at high risk of developing invasive candidosis.** IC, invasive candidosis.

From this kinetic analysis, we propose an algorithm (Figure 4) for preemptive treatment based on the principle of biological screening of ICU populations, the majority of whom are at high risk of IC. BDG detection is a more sensitive, early positive test, and therefore appears useful for first-line screening. For BDG values >800 pg/ml, specificity values >90% require no confirmatory test. For a cutoff value <800 pg/ml and >80 pg/ml, determination of mannan is indicated since the high correlation between a combination of these two positive biomarkers and IC could be an indication for preemptive treatment. In other cases, regular monitoring of glucanaemia is indicated since specificity increases by considering two or more successive positive results [13,39] and the cost of serological tests is minimal compared with antifungal treatment administered at excess. The colonisation index should be proposed as a last step despite its usefulness, due to the limitations of time constraints and cost.

The retrospective application of this sequential prescription of BDG and mannan detection in our cohort would have led to the treatment of 24 patients on the basis of BDG values >800 pg/ml and of five patients with BDG levels between 80 and 800 pg/ml associated with positive mannanaemia (total 29 patients = 70% of patients). When considering early positivity, more than 50% of the patients (22/41) would have been treated before positive BCs. Among the controls, 10 had positive BDG and four had positive mannan (14/67 = 15%). These results from the application of our biomarker-based algorithm for preemptive antifungal therapy compare favourably with empiric treatments, which have been shown not to improve the outcome in cases of fluconazole prescription [40] or to be cost-effective [41]. Similarly, the results compare favourably with probabilist score-based therapy. Indeed, the clinical prediction rule developed by Ostrosky-Zeichner and colleagues captured 10.6% of the patients with proven/probable IC, but also 34.1% of patients without IC [42]; the *Candida* score from Leon and colleagues exhibited an area under the receiver operating characteristic curve of 0.774, a sensitivity of 77.6 and a specificity of 66.2 [43]. A limitation to this approach is that laboratory procedures often prevent individual testing and, despite the fact that only 2 to 3 hours are needed to obtain the results, sera are often treated as a series, once or twice a week. The periodicity of testing should be adapted for institutions with small numbers of samples or the tests should be performed in a reference centre.

The data presented and discussed here suggest that, due to the lack of sensitivity of BCs, managing therapeutic decisions using the *Candida* glycan detection test is not unrealistic now that tests are able to detect molecules in the range of picogrammes per millilitre with high reliability. New more sensitive methods for the detection of glycan biomarkers will probably be developed, establishing a panel of tests that cannot be ignored for patient care, especially in the ICU.

## Conclusion

An algorithm for preemptive therapy based on a combination of BDG and mannan detection was derived from a kinetic analysis of a large volume of clinical and biological data collected from patients hospitalised in the same ward and who developed candidaemia or not. The follow-up of BDG and mannan kinetics may also predict relapses. This strategy, which could be adapted to the management of the large number of ICU patients with negative *Candida* BCs, should be validated through large prospective studies.

## Key messages

• Detection of *Candida* cell wall polysaccharides in serum is a useful adjunct to BCs for the diagnosis of IC in the ICU.

• The kinetics and duration of circulation differ between BDG and mannan and between infected patients and colonised controls: BDG is an early sensitive biomarker but has low specificity, while mannan appears later and is less sensitive, but has high specificity.

• Increasing the cutoff value for BDG and combining BDG detection with mannan detection can help in preemptive therapy.

• Mannan and BDG follow-up is not useful for monitoring treatment efficacy, but an increase in these markers is predictive of relapse.

## Abbreviations

Ab: antibody; BC: blood culture; BDG: β-d-1,3-glucan; IC: invasive candidosis.

## Competing interests

DP and BS received research grants from Bio-Rad. The remaining authors declare that they have no competing interests.

## Authors' contributions

JP collected data, performed the biological tests, performed the statistical analysis and wrote the paper. BS helped with the selection of sera, helped in the interpretation of the results and wrote the paper. SD participated in the experiments and gave critical analysis in the interpretation of the results. KII participated in the experiments and the analysis of the results. NF helped with the selection of sera from the serum bank and with the biological tests. MK helped to collect clinical data. RF advised on the statistical analysis. DM shared his experience in patient management and helped with the statistical analysis and the development of the algorithm. DP coordinated the work, gave critical advice on the interpretation of the results and wrote the manuscript. All authors read and approved the final manuscript.

## Supplementary Material

Additional file 1 **is a clinical description of patients with relapses of candidaemia, and for whom kinetics profiles of biomarkers are shown in Figure** 2**.** Click here for file

Additional file 2 is a graphical representation of the distribution of glucanaemia reported to the daily fungal load, showing there is no correlation between colonisation and glucanaemia. Click here for file

Additional file 3 **is a graphical representation of the relation between mean global *Candida *** **load and glucanaemia, showing a trend for an association between a cumulative high level of colonisation during all of the ICU stay and a high glucanaemia level.** Click here for file
