# Supplementary material for: Presence of Candida cell wall derived polysaccharides in the sera of intensive care unit patients: relation with candidaemia and Candida colonisation
Source: Crit Care. 2014 Jun 29;18(3):R135. doi: 10.1186/cc13953 (PMC4227034; doi:10.1186/cc13953)
Supplement: Additional file 1 — is a clinical description of patients with relapses of candidaemia, and for whom kinetics profiles of biomarkers are shown in Figure 2. [file cc13953-S1.docx]

**Supplementary material**

***Clinical description of patients having presented relapses and for whom kinetic profiles of BDG, mannans and anti-mannans antibodies are shown on figure 2.***

Patient 1 was hospitalized for a septic shock due to a peritonitis secondary to ischemic colitis and sigmoidal perforation, in a context of auriculo-ventricular dysfonction. Candidemia occurred ten days after Hartman operation, while colic resection and splenectomy were needed due to ischemia. The patient remained candidemic despite antifungal therapy and died 15 days after the first episode of candidemia.

Patient 2 had CO poisoning and burns, had a short corticotherapy for an ARDS tracheotomy and was under mechanical ventilation during 16 days. This patient survived.

Patient 3 was hospitalized for septic shock in a context of neutropenia secondary to an hematopoietic stem cell transplant for an idiopathic medullar aplasia, remained neutropenic during all its hospitalization, and developed the first candidemia despite prophylactic antifungal therapy, died in a context of hemorrhagic cerebral stroke, with a suspicion of endocarditis.

Patient 4 hospitalized for an ARDS and a septic shock secondary to a pneumonia due to *Legionnella pneumophila*, had vasoactive support by noradrenaline, activated protein C administration, veno-venous discontinued hemodiafiltration, the first blood culture positive was sampled 48 hours after an antifugal treatment by echinocandin was instituted. This patient was discharged alive from ICU.

Patient 5 suffered from chronic obstructive bronchopneumonia and diabetes mellitus was hospitalized in ICU for a septic shock due to diabetic foot ulcer, had veno-venous discontinued hemodiafiltration, vasoactive support by noaradrenaline and dobutamine, as well as activated protein C administration. This patient was discharged alive from ICU.
