# Supplementary figures and images for: Presence of Candida cell wall derived polysaccharides in the sera of intensive care unit patients: relation with candidaemia and Candida colonisation
Source: Crit Care. 2014 Jun 29;18(3):R135. doi: 10.1186/cc13953 (PMC4227034; doi:10.1186/cc13953)

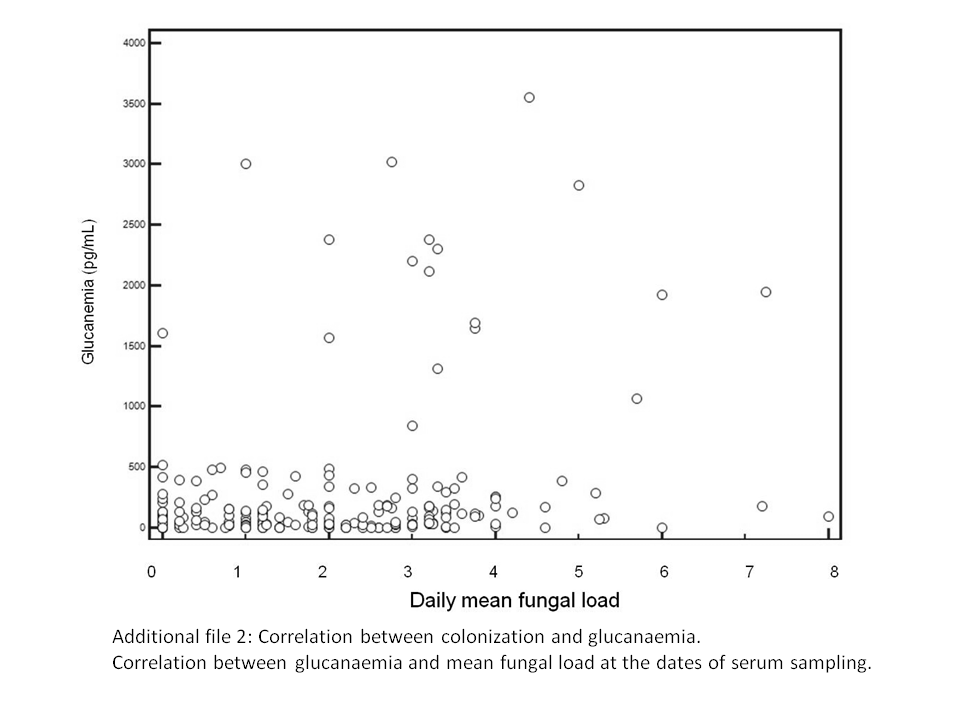

Supplement: Additional file 2 — is a graphical representation of the distribution of glucanaemia reported to the daily fungal load, showing there is no correlation between colonisation and glucanaemia. [file cc13953-S2.tiff]

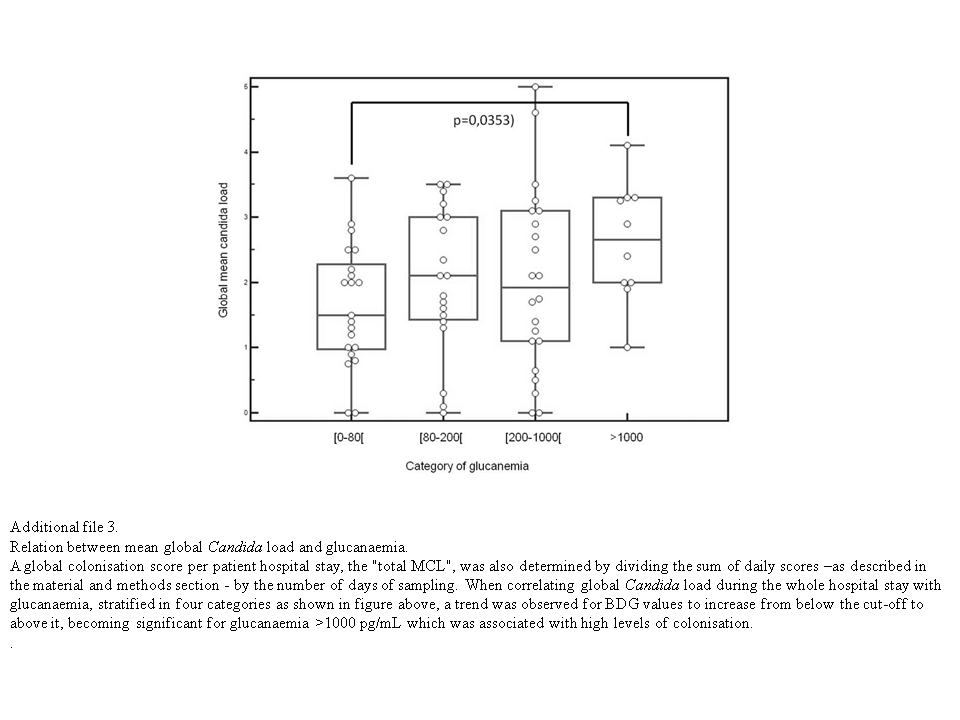

Supplement: Additional file 3 — is a graphical representation of the relation between mean global Candida load and glucanaemia, showing a trend for an association between a cumulative high level of colonisation during all of the ICU stay and a high glucanaemia level. [file cc13953-S3.tiff]
